# Focused Cardiac Ultrasound Curriculum for Internal Medicine Residents

**DOI:** 10.24908/pocus.v6i1.14759

**Published:** 2021-04-22

**Authors:** Dipika Gopal, Cameron Baston, Srinath Adusumalli, Dinesh Jagasia, Stuart Prenner

**Affiliations:** 1 Division of Cardiovascular Medicine, University of Pennsylvania Perelman School of Medicine Philadelphia, Pennsylvania; 2 Division of Pulmonary & Critical Care, University of Pennsylvania Perelman School of Medicine Philadelphia, Pennsylvania

**Keywords:** focused cardiac ultrasound, curriculum assessment, point-of-care ultrasound, POCUS

## Abstract

**Background: **Focused cardiac ultrasound (FCU) is a safe and efficient diagnostic intervention for internal medicine physicians. FCU is a highly teachable skill, but is used in routine cardiac assessment in only 20% of surveyed training programs.We developed an FCU curriculum for internal medicine residents and an assessment tool to evaluate the impact of the curriculum on trainee knowledge and confidence. **Methods: **Internal medicine residents rotating through clinical cardiology services underwent 30 minutes of didactic and 60 minutes of hands-on teaching on acquisition and interpretation of FCU. A 20 item pre and post-curriculum online survey was administered (November 2018-December 2019) to assess confidence and knowledge in FCU. **Results: **79 of 116 (68%) residents completed the pre-survey and 50 completed the post-survey, of whom 34 received the curriculum. The mean change in confidence score in those who received versus did not receive the curriculum was 0.99 versus 0.39 (p=0.046) on a 5-point Likert scale. Among 33 residents who had paired pre- and post-surveys the mean change in confidence score was 1.2 versus 0.85 (p<0.001) in those who received versus did not receive the curriculum. The mean increase in knowledge score was 13% versus 7% respectively (p<0.0001). **Conclusions: **We instituted a novel curriculum for internal medicine residents to gain experience in image acquisition and interpretation. Both confidence and knowledge in FCU improved following the curriculum, indicating that this is a highly teachable skill. Additional analysis of the of the FCU study images will be useful for informing future interventions.

## Background

Focused cardiac ultrasound (FCU) has gained immense traction within health systems as an opportunity for a safe, efficient, and cost-effective diagnostic tool 1, 2, 3. In an era of digitization and speed, the physical examination is being complemented by a more comprehensive and accurate sonographic cardiovascular assessment 4. 

Several studies show that FCU is a highly teachable skill at all educational levels including medical students, residents, and hospitalists 5, 6, 7. Brief training modules can prepare residents sufficiently to diagnose left ventricular systolic dysfunction on images they obtain and interpret compared to transthoracic echocardiogram images interpreted by a level 3 trained echocardiographer 5. Handheld ultrasounds allow physicians to detect pathology in a timely manner and potentially decrease downstream cost of unnecessary testing 6. Interestingly, abnormalities detected on FCU have also been shown to predict readmission for heart failure better than any of the standard risk factors including patient comorbidities and biomarkers 7. 

The Accreditation Council for Graduate Medical Education states that internal medicine residents should be exposed to sonography to develop competency in bedside sonography, however specific training methodologies are not published 8. Trainees commonly miss critical cardiac pathological findings and misjudge volume status based on visual jugular venous pressure assessment 9. Numerous studies, however, have shown that trainees can learn bedside ultrasonography to arrive at accurate clinical assessments 5, 6, 10, 11, 12. Despite this, a recent national survey of medical education leaders revealed that in routine clinical practice cardiac and volume assessment using bedside sonography was done in 20% of training programs even though the perceived usefulness was rated 3.9 on a 5 point scale 13. In that same study, only 25% of the programs reported having a formal curriculum 1. 

Though several informal curriculums exist, there is sparse data demonstrating the quantitative impact of such curriculums on medical residents.^14^, ^15^ We developed an FCU curriculum for internal medicine residents and an assessment tool to evaluate resident knowledge of FCU as well as confidence in image acquisition and interpretation.

## Methods

The inpatient cardiology rotations at our institution consist of the cardiac care unit and the cardiac intermediate care unit where four residents rotate on each of these services for two weeks at a time. Our FCU curriculum was embedded into the longitudinal cardiology curriculum already in existence for these rotations. The curriculum consisted of a 30-minute didactic session focusing on principles of cardiac ultrasound, instrumentation, image acquisition, optimization, and interpretation and a 60-minute precepted hands-on session where residents practiced image acquisition on patients admitted under their care guided by a facilitator. Two devices, the Sonosite Xporte and the Sonosite EDGE 2 (FUJIFILM, Washington, USA) were used to practice all image acquisition. The curriculum was administered or withheld on alternating 2-week blocks to generate two cohorts of residents: those who received and those who did not receive the curriculum.

Residents completed pre-curriculum and post-curriculum surveys that were developed by our research group assessing their confidence and fund of knowledge in image acquisition, interpretation, and clinical integration of findings (online supplementary Table S1). Confidence was assessed on a 5-point Likert scale (1-Not at all confident, 2-Only slightly confident, 3-Somewhat confident, 4-Moderately confident, 5-Very confident). If a resident received the curriculum more than once we used only the first paired pre- and post-curriculum surveys for analysis. In order to minimize contamination, we eliminated all duplicate unique identifiers and those who reported taking the curriculum chronologically before the pre-curriculum survey. Change in confidence and percent correct on the knowledge questions was analyzed via a paired t-test. P value was set at 0.05 for significance.

## Results

Seventy-nine of 116 (68%) residents who rotated through cardiology between November 2018 and December 2019 completed the pre-curriculum survey and 50 residents completed the post-curriculum survey. Of those 50, 34 residents reported having received the didactic and hands-on curriculum. The residents were evenly distributed among PGY level. 58% (46/79) had never received any form of Point of Care Ultrasound (POCUS) curriculum prior to the study (Table 1). The mean confidence score increased by 0.80 on a 5-point Likert scale and the mean knowledge score increased by 7% in the total population. Among those who received the curriculum, the mean change in confidence score was 0.99 compared to 0.39 in those who did not receive the curriculum (p=0.05, Table 2). The mean change in knowledge score from pre- to post-survey was not significantly different in the two groups (8.7% in the group that did receive the curriculum compared to 9.2% in the group that did not receive the curriculum, p=0.88, Table 2).

**Table 1:** Demographics

**Demographics**	
PGY Level	
1	25 (32%)
2	26 (32%)
3	21 (27%)
Not Stated	7 (9%)
Prior POCUS Curriculum	
none	46 (58%)
lectures only	7 (9%)
hands-on only	5 (6%)
lectures and hands-on	14 (18%)
Not Stated	7 (9%)

**Table 2:** Confidence and Knowledge Scores

**Total Study Population**				**Matched pre- and post-assessment Population**		
	**Curriculum (n=34)**	**No Curriculum (n=16)**	**P value**	**Curriculum (n=25)**	**No Curriculum (n=8)**	**P value**
Mean change in confidence score	0.99	0.39	0.046	1.20	0.85	<0.001
Mean change in knowledge score	8.7%	9.2%	0.880	13.0%	7.0%	<0.001

Thirty-three residents took both the pre and post-surveys, of whom 76% (25/33) stated that they had received the curriculum. There was a 13% mean increase in knowledge score in the group that received the curriculum compared to 7% increase in the group that did not receive the curriculum (p<0.001, Table 2). While confidence scores improved regardless of whether the resident had received the curriculum, there was a statistically significant greater increase in mean confidence scores among residents who received the curriculum versus not (1.20 vs 0.85, p<0.001, Table 2).

For the confidence assessment, two questions in the acquisition domain were identified as most able to discriminate whether the resident had received the curriculum. The mean confidence score in optimization of image quality with knob manipulation including gain and depth decreased (3.3 to 3.2) in those who did not receive the curriculum and increased in those who did (3.3 to 3.5). Residents who had the curriculum experienced a greater increase in confidence in correctly identifying cardiac anatomy compared to residents who did not receive the curriculum (3.0 to 3.5 vs 3.0 to 3.1).

## Discussion

This study adds to the growing body of literature on the development and assessment of FCU curricula. Our results demonstrate that internal medicine residents at our institution have a high fund of knowledge in FCU interpretation, albeit with some heterogeneity. Confidence and skill in image acquisition were incrementally improved with the addition of a hands-on curriculum.

Although the mean knowledge score improved in all residents, the subset of residents who had matched pre- and post-survey data showed that there was a statistically significant higher increase in score in the group that received the curriculum. This speaks to the benefit of an FCU curriculum to address specific knowledge gaps and provide a space in which to clarify questions with FCU experts on material residents may have learned through clinical practice. This may have also occurred because the didactic portion of the curriculum provided a dedicated space to learn the material in close proximity to the survey, increasing scores overall in that group. This is consistent with existing literature showing that bedside ultrasound (cardiac, lung abdomen, etc.) is a teachable skill to internal medicine residents 2, 3. 

It has been shown in several POCUS curricula that hands-on teaching improves competency and confidence in independent image acquisition and interpretation 2, 3, 4. We re-demonstrated this in our study among a population of highly trained internal medicine residents already receiving clinical teaching on FCU. After a short didactic and hands-on session, residents felt substantially more comfortable in optimizing images with knob manipulation and identification of cardiac anatomy. Whether the skills acquired in the hands-on portion of the curriculum translate into improved FCU images acquired subsequently in clinical care is a worthwhile research question to pursue. 

The pre- and post-curriculum assessment tool is a novel addition to existing literature as it provided a way of discriminating between those who did and did not receive the curriculum. While there exist competency assessments for individualized curriculums, our tool remains purposefully broad to encompass the basic tenets any FCU curriculum would cover thus enhancing its external validity.

Several limitations should be noted. This study was conducted at a large single center academic internal medicine residency program and thus repeated studies are warranted to ensure external validity. Importantly, high pre-curriculum knowledge scores indicate that residents who rotate through cardiology benefit from existing methods of ultrasound teaching on rounds and during clinical care. Thus, the benefit of the intervention in this study may be more attenuated than in other programs. Resident self-reporting may have limited the impact assessment of our curriculum. Residents who attended the curriculum were more likely to fill out a post-curriculum survey, and there was drop off in participation between the pre- and post-surveys. Despite these limitations, our study successfully demonstrated the additive benefit of a hands-on curriculum in teaching FCU to internal medicine residents. Future studies will help us understand the endurance of the curriculum, retention of knowledge and skill, and the effect it may have on clinical care.

## Conclusion

We instituted a novel curriculum for internal medicine residents to learn FCU and gain experience in image acquisition and interpretation resulting in improvement in confidence and knowledge in FCU.

## Statement of ethics approval/consent:

This study was approved by the Penn Institutional Review Board and each resident provided informed consent included in the online survey to participate in the study.

## Disclosures

The authors declare no conflicts of interest.

## Supplementary Material

Table S1 Confidence and Knowledge Questions
