# Supplementary material for: Focused Cardiac Ultrasound Curriculum for Internal Medicine Residents
Source: POCUS J. 2021 Apr 22;6(1):29–32. doi: 10.24908/pocus.v6i1.14759 (PMC9979930; doi:10.24908/pocus.v6i1.14759)
Supplement: Table S1 [file pocusj-06-14759-s001.pdf]

Table S1: Confidence and Knowledge Questions

Please rate your confidence to acquire the following images/answer the following clinical questions on a scale of 1-5 with 1 being Not at all confident and 5 being Very confident	
	Optimize image quality with knob manipulation including gain and depth.
	Determine whether the left ventricle is dilated or normal size.
	Determine whether left ventricular function is grossly normal or abnormal.
	Determine whether the right ventricle is dilated or normal size.
	Determine whether an effusion near the heart is pleural or pericardial.
	Determine whether a patient is likely to have high right atrial pressure based on IVC measurements.
	Determine whether signs of a recent acute pulmonary embolism are present in a patient with previously normal heart.
	Identify all cardiac chambers and valves in multiple cardiac views.
Please answer the following interpretation questions to the best of your ability.	
	From which imaging location is the following image obtained?
	What is the best clinical interpretation of the following two clips?
	From which imaging location is the following image obtained?
	Where should the echo probe be placed to obtain a parasternal long axis (PLAX) image?
	From which imaging location is the following image obtained?
	How should the echo probe be manipulated to go from a parasternal long axis image to a short axis image?
	Identify the right ventricle in the following view
	From which imaging location is the following image obtained?
	Identify the left atrium in the following view
	Based on the following clip, what do you estimate the right atrial pressure to be in this spontaneously breathing patient?
	Based on the following clip, what do you estimate the right atrial pressure to be in this spontaneously breathing patient?
	What is the best clinical interpretation of the following clip?
	What structure should the indicator dot on the probe be facing in the subcostal four chamber view?
	What is the best clinical interpretation of the following clip?
	Which of the following 2-D clips of the IVC could support that a patient with a pericardial effusion might have tamponade?
	In this patient with chest pain and shock, based on the following two videos what is the most likely diagnosis?
	In this patient with chest pain and shock, based on the following video, what is the most likely diagnosis?

Link to pre-curriculum survey: https://redcap.link/81yp32xu
